# Preoperative albumin to globulin ratio predicts survival in clear cell renal cell carcinoma patients

**DOI:** 10.18632/oncotarget.15162

**Published:** 2017-02-07

**Authors:** Zhen Chen, Yingjie Shao, Hongwei Yao, Qianfeng Zhuang, Kun Wang, Zhaoyu Xing, Xianlin Xu, Xiaozhou He, Renfang Xu

**Affiliations:** ^1^ Department of Urology, The Third Affiliated Hospital of Soochow University, Changzhou, P.R. China; ^2^ Department of Radiation Oncology, The Third Affiliated Hospital of Soochow University, Changzhou, P.R. China; ^3^ Department of Urology, Sir Run Run Shaw Hospital, Third Affiliated Hospital, Nanjing Medical University, Nanjing, Jiangsu, P.R. China

**Keywords:** albumin, globulin, nomogram, prognosis, renal cell carcinoma

## Abstract

In this retrospective analysis, we evaluated associations between albumin to globulin ratio (AGR), clinicopathological characteristics, and survival in 592 patients with localized or locally advanced clear cell renal cell carcinoma (CCRCC) prior to nephrectomy. We found that low AGR was associated with more aggressive tumor behavior; patients with low AGR had poorer overall survival (OS) and cancer-specific survival (CSS) in Kaplan-Meier survival analyses both before and after propensity score matching, which was used to compensate for differences in baseline clinicopathological characteristics. AGR was an independent prognostic factor for both OS (HR: 6.799; 95% CI: 3.215−14.377; *P* < 0.001) and CSS (HR: 8.806; 95% CI: 3.891−19.928; *P* < 0.001), and its prognostic value was higher than that of other established inflammation-based prognostic scores. When AGR was incorporated into a prognostic model that included T stage, neutrophil to lymphocyte ratio (NLR), and monocyte to lymphocyte ratio (MLR), the resulting nomogram predicted 3- and 5-year OS in the patients more accurately than when AGR was not included. In conclusion, AGR may be particularly useful for improving clinical outcome predictions for patients with localized or locally advanced CCRCC.

## INTRODUCTION

Renal cell carcinoma (RCC) accounts for 2%–3% of adult malignancies, and is the second most common type of urogenital neoplasm after bladder cancer [1]. Clear cell renal cell carcinoma (CCRCC) is the most common pathological subtype, accounting for 70%–80% of all RCC cases. As RCC is not sensitive to chemoradiotherapy, radical nephrectomy remains the most promising treatment for patients with localized or locally advanced RCC. However, postoperative recurrence rates of 20%–30% are observed after this treatment [2]. In order to evaluate postoperative risks and to improve individualized treatment, several prognostic models based on clinicopathological characteristics, such as TNM stage [3] and Fuhrman grade [4], have been established to predict outcomes in RCC patients. However, prognostic models based on clinicopathological characteristics alone are less effective for patients with localized or locally advanced CCRCC; a novel prognostic model is urgently needed to improve outcome predictions for these patients.

Albumin and globulin are major serum proteins, and low albumin and high globulin levels reflect not only malnutrition, but also a chronic inflammatory state in the body [5, 6]. The albumin to globulin ratio (AGR) better reflects the nutritional and inflammatory state by combining these two indicators in one measure. Inflammatory reactions associated with malnutrition greatly reduce immune system function, alter the tumor microenvironment, promote tumor growth and metastasis, and negatively impact prognosis in cancer patients [7, 8]. Accordingly, AGR seems to be indicative of disease progress in cancer. Several studies have confirmed that AGR is associated with prognosis in breast [9], colorectal [10], nasopharyngeal [11], and lung cancers [12]. However, the association between AGR and prognosis in RCC patients has not yet been examined.

In this paper, we evaluated the prognostic value of preoperative AGR and compared its value to that of other inflammation-based prognostic scores. Furthermore, we identified a nomogram that combines inflammation-based prognostic scores with clinicopathological characteristics to accurately predict 3- and 5-year overall survival (OS) in patients with localized or locally advanced CCRCC after radical or partial nephrectomy.

## RESULTS

### Patient characteristics

The clinicopathologic characteristics of patients in the primary and validation cohorts are shown in Table 1. The primary cohort consisted of a total of 258 men (62.0%) and 158 women (38.0%) with localized or locally advanced CCRCC who underwent radical or partial nephrectomy. 154 (37.0%) of these patients were older than 60 years, and 262 (63.0%) were younger; the median age was 56.3 (range 24–80) years. The median follow-up period was 69.2 (range 1–151) months; at final follow-up, 51 (12.3%) patients had died, and 365 (87.7%) were alive.

**Table 1:** Characteristics of primary and validation cohort patients

Variable	Primary Cohort (*n* = 416)	Validation cohort (*n* = 176)
Age (years)		
≤ 60	261 (62.9 %)	105 (59.7 %)
> 60	154 (37.1 %)	71 (40.3 %)
Sex		
Male	258 (62.2 %)	115 (65.3 %)
Female	157 (37.8 %)	61 (34.7 %)
T stage		
1	337 (81.6 %)	144 (81.8 %)
2	44 (10.7 %)	18 (10.2 %)
3	32 (7.7 %)	14 (8.0 %)
N stage		
0	405 (97.6 %)	174 (98.9 %)
1	10 (2.4 %)	2 (1.1 %)
Fuhrman grade		
1	85 (21.1 %)	34 (19.3 %)
2	200 (49.8 %)	80 (45.5 %)
3	94 (23.4 %)	43 (25.0 %)
4	23 (5.7 %)	10 (5.7 %)
Tumor size, cm		
≤ 5	265 (64.2 %)	114 (64.8 %)
> 5	148 (35.8 %)	61 (34.7 %)
Tumor necrosis		
Absent	375 (90.4 %)	158 (89.8 %)
Present	40 (9.6 %)	18 (10.2 %)
LVI		
Absent	394 (94.9 %)	165 (93.8 %)
Present	21 (5.1 %)	11 (6.3 %)
Hemoglobin (g/L)		
≤ LLN	49 (11.9 %)	20 (11.4 %)
> LLN	364 (88.1 %)	156 (88.6 %)
AKP		
≤ ULN	396 (96.4 %)	171 (97.1 %)
> ULN	15 (3.6 %)	5 (2.9 %)
LDH		
≤ 1.5*ULN	404 (98.3 %)	174 (98.8 %)
> 1.5*ULN	7 (1.7 %)	2 (1.2 %)
BMI (Kg/ m^2^)		
≤ 18.5	43 (10.3 %)	26 (14.8 %)
18.5–24.5	313 (75.2 %)	99 (56.2 %)
> 24.5	56 (13.5 %)	49 (27.8 %)
AGR		
**≤ 1.22**	72 (17.3)	32 (18.2)
**> 1.22**	344 (82.7)	144 (81.8)
NLR		
≤ 2.17	214 (51.7 %)	96 (54.5 %)
> 2.17	199 (48.2 %)	80 (45.5 %)
MLR		
≤ 0.30	273 (66.1 %)	118 (67.0 %)
> 0.30	140 (33.9 %)	58 (33.0 %)
PLR		
≤ 179.83	356 (86.0 %)	153 (86.9 %)
> 179.83	57 (13.8 %)	22 (12.5 %)
GPS		
0	351 (84.6 %)	149 (84.7)
1	46 (11.1 %)	16 (9.1)
2	18 (4.3 %)	11 (6.3)
mGPS		
0	366 (88.6 %)	153 (86.9 %)
1	29 (7.0 %)	12 (6.8 %)
2	18 (4.4 %)	11 (6.3 %)

Abbreviations: PSM, propensity score matching; LVI, lymphovascular invasion; AKP, alkaline phosphatase; LDH, lactate dehydrogenase; BMI, body mass index; AGR, albumin to globulin ratio; NLR, neutrophil to lymphocyte ratio; MLR, monocyte to lymphocyte ratio; PLR, the platelet to lymphocyte ratio; GPS, Glasgow Prognostic Score; mGPS, modified Glasgow Prognostic Score.

The validation cohort included 115 men (65.3%) and 61 women (34.7%); 71 (40.3%) of these patients were older than 60 years. The median follow-up period was 42.3 (range 3–50) months. At final follow-up, 23 (13.1%) patients had died, and 153 (86.9%) were alive.

### Cut-off values for continuous variables

The cut-off values for all continuous variables are shown in Table 1. The optimal cut-off value of 1.22 for AGR was used to divide patients into two groups (≤ 1.22, *n* = 71; > 1.22, *n* = 344). Optimal cut-off values for neutrophil to lymphocyte ratio (NLR), monocyte to lymphocyte ratio (MLR), and platelet to lymphocyte ratio (PLR) were 2.17, 0.30, and 179.83, respectively. Lower limits of the normal range, which were 130 g/L for males and 115 g/L for females, were used as cut-off values for hemoglobin (Hb). The upper limit of the normal range, 125 U/L, was used as the cut-off value for alkaline phosphatase (ALP), while 1.5 times the upper limit of 245 U/L was used for lactate dehydrogenase (LDH).

### Associations between AGR and primary cohort patient clinicopathological characteristics

Associations between AGR and clinicopathological characteristics in the 416 primary cohort patients are shown in Table 2. Low AGR was associated with older age at surgery (*P* < 0.001), higher T stage (*P* < 0.001), *N* stage (*P* = 0.005), and Fuhrman grade (*P* = 0.001), larger tumor size (*P* < 0.001), the presence of tumor necrosis (*P* < 0.001) and lymphovascular invasion (*P* = 0.001), lower Hb concentration (*P* < 0.001), higher AKP (*P* < 0.001) and LDH (*P* = 0.005) concentrations, and lower BMI (*P* < 0.001).

**Table 2:** Associations between AGR and clinicopathological characteristics in primary cohort patients before and after PSM

Variable	Pre-PSM			Post-PSM		
	AGR ≤ 1.22 (*n* = 72)	AGR > 1.22 (*n* = 344)	*P* value	AGR ≤ 1.22 (*n* = 52)	AGR > 1.22 (*n* = 52)	*P* value
Age (years)			< 0.001*			0.695
≤ 60	29 (40.8 %)	232 (67.4 %)		24 (46.2 %)	26 (50.0 %)	
> 60	42 (59.2 %)	112 (32.6 %)		28 (53.8 %)	26 (50.0 %)	
Sex			0.399			0.543
Male	41 (57.7 %)	217 (63.1 %)		31 (59.6 %)	34 (65.4 %)	
Female	30 (42.3 %)	127 (36.9 %)		21 (40.4 %)	18 (34.3 %)	
T stage			< 0.001*			
1	42 (59.2 %)	295 (86.3 %)		40 (76.9 %)	37 (71.1 %)	0.468
2	12 (16.9 %)	32 (9.4 %)		8 (15.4 %)	7 (13.5 %)	
3	17 (23.9 %)	15 (4.4 %)		4 (7.7 %)	8 (15.4 %)	
N stage			0.005*			
0	66 (93.0 %)	339 (98.5 %)		50 (96.2 %)	50 (96.2 %)	1
1	5 (7.0 %)	5 (1.5 %)		2 (3.8 %)	2 (3.8 %)	
Fuhrman grade						
1	8 (11.8 %)	77 (23.1 %)		6 (13.3 %)	1 (2.2 %)	
2	30 (44.1 %)	170 (50.9 %)	0.001*	25 (55.6 %)	25 (55.6 %)	0.228
3	20 (29.4 %)	74 (22.2 %)		11 (24.4 %)	15 (33.3 %)	
4	10 (14.7 %)	13 (3.9 %)		3 (6.7 %)	4 (8.9 %)	
Tumor size, cm						
≤ 5	30 (42.9 %)	235 (68.5 %)	< 0.001*	28 (53.8 %)	31 (59.6 %)	0.553
> 5	40 (57.1 %)	108 (31.5 %)		24 (46.2 %)	21 (40.4 %)	
Tumor necrosis			< 0.001*			0.426
Absent	56 (78.9 %)	319 (92.7 %)		45 (86.5 %)	42 (80.8 %)	
Present	15 (21.1 %)	25 (7.3 %)		7 (13.5 %)	10 (19.2 %)	
LVI			0.001*			0.462
Absent	62 (87.3 %)	332 (96.5 %)		49 (94.2 %)	47 (90.4 %)	
Present	9 (12.7 %)	12 (3.5 %)		3 (5.8 %)	5 (9.6 %)	
Hemoglobin (g/L)			< 0.001*			0.631
≤ LLN	27 (38.6 %)	22 (6.4 %)		12 (23.1 %)	10 (19.2 %)	
> LLN	43 (61.4 %)	321 (93.6 %)		40 (76.9 %)	42 (80.8 %)	
AKP			< 0.001*			1
≤ ULN	62 (87.3 %)	334 (98.2 %)		48 (92.3 %)	48 (92.3 %)	
> ULN	9 (12.7 %)	6 (1.8 %)		4 (7.7 %)	4 (7.7 %)	
LDH			0.005*			0.315
≤ 1.5*ULN	67 (94.4 %)	337 (99.1 %)		52 (100.0 %)	51 (98.1 %)	
> 1.5*ULN	4 (5.6 %)	3 (0.9 %)		0 (0.0 %)	1 (1.9 %)	
BMI (Kg/ m^2^)						
≤ 18.5	28 (38.9 %)	15 (4.4 %)		22(42.3 %)	13 (25.0 %)	
18.5–24.5	29 (40.3 %)	284 (82.5 %)	< 0.001*	20 (38.5 %)	29 (55.8 %)	0.138
> 24.5	14 (19.4 %)	42 (12.2 %)		10 (19.2 %)	10 (19.2 %)	

* *p* < 0.05

Abbreviations: PSM, propensity score matching; LLN, lower limit of normal; ULN, upper limit of normal; LVI, lymphovascular invasion; AKP, alkaline phosphatase; LDH, lactate dehydrogenase; BMI, body mass index; AGR, albumin to globulin ratio.

### Prognostic value of AGR

**Figure 1:**
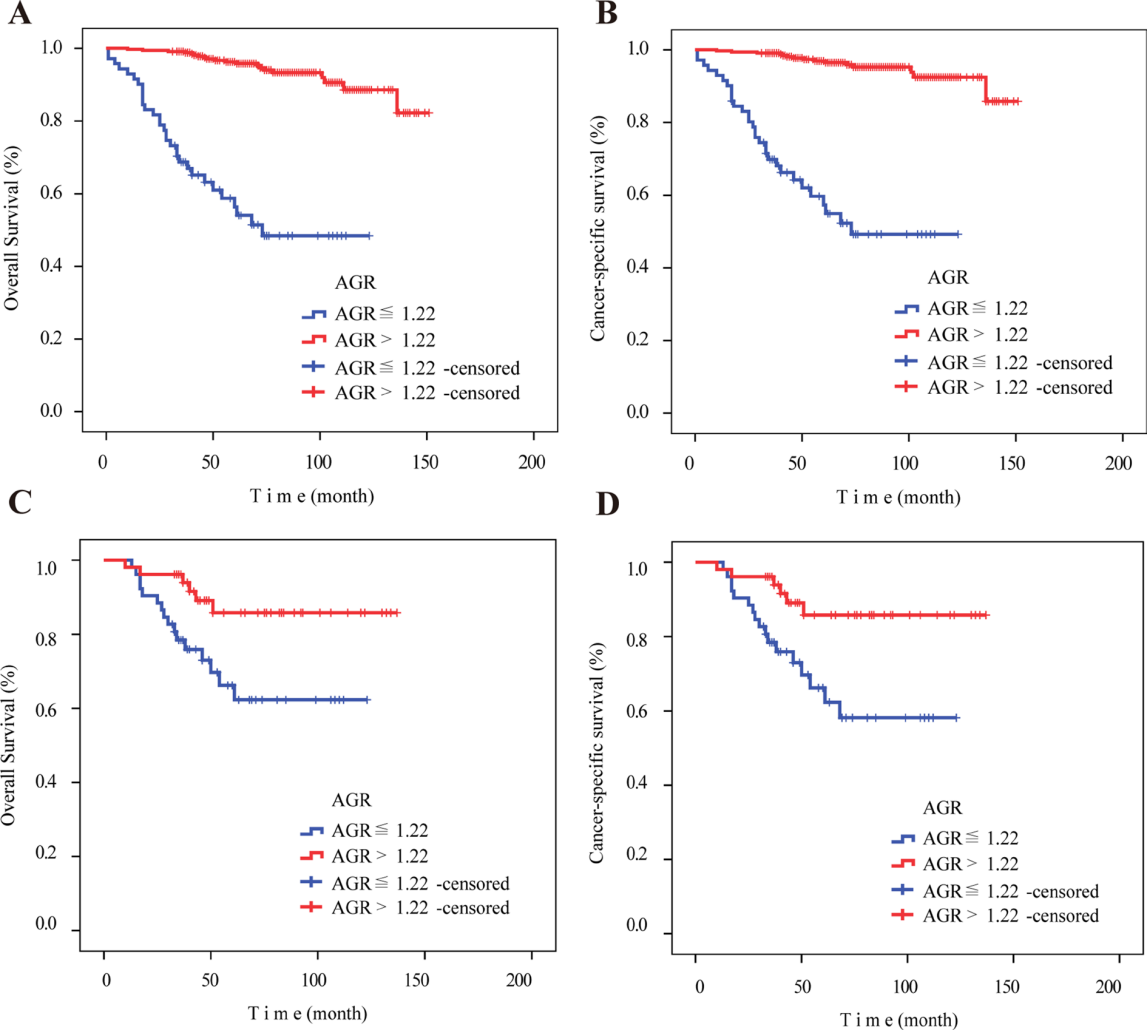
Kaplan–Meier curves for overall survival and cancer-specific survival according to preoperative AGR before and after PSM (**A**) Overall survival (*P* < 0.001) and (**B**) cancer-specific survival (*P* < 0.001) were worse in patients with low AGR (≤ 1.22) before PSM. (**C**) Overall survival (*P* = 0.012) and (**D**) cancer-specific survival (*P* = 0.007) were worse in patients with low AGR (≤ 1.22) after PSM. Abbreviation: AGR, albumin to globulin ratio. PSM, propensity score matching.

OS (*P* < 0.001) and CSS (*P* < 0.001) were worse in patients with low AGRs (≤ 1.22) than in patients with high AGRs (> 1.22) prior to propensity score matching (PSM) (Figure 1A, 1B). Because some clinicopathological characteristics differed between the low and high AGR patient groups (Table 2), we performed PSM to minimize these differences. In the PSM analysis, 52 patients selected from the high AGR group were each paired with one low AGR patient using a nearest-neighbor algorithm. After PSM, clinicopathological characteristics were balanced and evenly distributed between the low and high AGR groups (all *P* > 0.05) (Table 2). Kaplan-Meier survival curves for the post-PSM low and high AGR groups confirmed that OS and CSS were still worse in low AGR patients, even when controlling for differences in clinicopathological characteristics (Figure 1C, 1D).

The results of the multivariate analysis for OS and CSS before PSM in the primary cohort are shown in Table 3. Because AGR, NLR, MLR, PLR, GPS, and mGPS were correlated initially, we then conducted multivariate analysis for these inflammation-based prognostic scores. AGR (HR: 6.529; 95% CI: 3.036–14.042; *P* < 0.001), NLR (*P* = 0.001), MLR (*P* = 0.001), GPS (*P* = 0.001), mGPS (*P* = 0.026), T stage (*P* = 0.005), and N stage (*P* = 0.041) were independently associated with OS. However, PLR (*P* = 0.909) was not an independent prognostic factor. The results of the multivariate analysis for CSS were very similar to those for OS.

**Table 3:** Overall survival, cancer-specific survival, and multivariate analyses in primary cohort patients before PSM

Variable	Multivariate analysis for OS		Multivariate analysis for CSS	
	HR (95 % CI)	*p* value	HR (95 % CI)	*p* value
Age(years)		0.323^a^		0.139 ^a^
> 60 years vs. ≤ 60 years	1.438 (0.700−2.957)		1.804 (0.825−3.941)	
Sex				
Male vs. Female				
T stage		0.005^* a^		0.003^* a^
1	reference		reference	
2	2.188 (0.874−5.480)	0.094 ^a^	2.548 (0.950−6.834)	0.063 ^a^
3	5.972 (2.049−17.407)	0.001^*a^	6.950 (2.298−21.021))	0.001^* a^
N stage		0.041^* a^		0.024^* a^
1 vs. 0	3.374 (1.050−10.846)		3.985 (1.203–13.024)	
Fuhrman grade		0.156 ^a^		0.140 ^a^
1	reference		reference	
2	2.037 (0.569−7.300)	0.274 ^a^	1.441 (0.384−5.401)	0.588 ^a^
3	2.119 (0.572−7.855)	0.261 ^a^	1.900 (0.498–7.250)	0.347 ^a^
4	0.617 (0.119−3.210)	0.566 ^a^	0.413 (0.075−2.294)	0.312 ^a^
Tumor size, cm		0.263 ^a^		0.564 ^a^
> 5 vs. ≤ 5	1.601 (0.703−3.646)		1.296 (0.538−3.120)	
Tumor necrosis		0.056^* a^		0.079 ^a^
Present vs. Absent	2.038 (0.982−4.226)		2.019 (0.922−4.423)	
LVI		0.127^a^		0.143 ^a^
Present vs. Absent	2.298 (0.790−6.682)		2.278 (0.757−6.856)	
Hemoglobin (g/L)		0.829^a^		0.674 ^a^
≤ LLN vs. > LLN	1.096 (0.476−2.528)		0.824 (0.336−2.025)	
AKP		0.507^a^		0.709 ^a^
> ULN vs. ≤ ULN	0.652 (0.184−2.306)		0.778 (0.208−2.911)	
LDH		0.824 ^a^		0.611 ^a^
> 1.5*ULN vs. ≤ 1.5*ULN	1.198 (0.244−5.887)		1.520 (0.303−7.624)	
AGR		< 0.0^01*a^		< 0.001^*a^
≤ 1.22 vs. > 1.22	6.529 (3.036–14.042)		8.806 (3.891–19.928)	
NLR		0.001^*b^		0.001^* b^
> 2.17 vs. ≤ 2.17	3.689 (1.752−7.766)		4.076 (1.805−9.203)	
MLR		0.001^*b^		0.004^* b^
> 0.30 vs. ≤ 0.30	3.406 (1.670−6.946)		2.961 (1.416−6.190)	
PLR		0.909 ^b^		0.828 ^b^
> 179.83 vs. ≤ 179.83	1.053 (0.435−2.545)		1.108 (0.439−2.795)	
GPS		0.001^*b^		0.003^* b^
0	Reference		Reference	
1	4.167 (2.000−8.684)	< 0.001^*b^	3.938 (1.799−8.618)	0.001^* b^
2	1.287 (0.372−4.455)	0.690b	1.579 (0.444–5.618)	0.481 ^b^
mGPS		0.026^*b^		0.158 ^b^
0	reference		reference	
1	3.156 (1.348−7.390)	0.008^*b^	2.537 (0.980−6.571)	0.055 ^b^
2	0.955 (0.286−3.189)	0.940^b^	1.164 (0.339−3.999)	0.809 ^b^

* *p* < 0.05

Abbreviations: PSM, propensity score matching; OS, overall survival; CSS, cancer-specific survival; LLN, lower limit of normal; ULN, upper limit of normal; AGR, albumin to globulin ratio; LVI, lymphovascular invasion; AKP, alkaline phosphatase; LDH, lactate dehydrogenase; NLR, neutrophil to lymphocyte ratio; MLR, monocyte to lymphocyte ratio; PLR, platelet to lymphocyte ratio; GPS, Glasgow Prognostic Score; mGPS, modified Glasgow Prognostic Score.

^a^ Age, T stage, N stage, Fuhrman grade, tumor size, tumor necrosis, LVI, hemoglobin, AKP, LDH, and AGR variables were tested in a multivariate analysis.

^b^ Established inflammation-based prognostic scores were evaluated together with age, T stage, N stage, Fuhrman grade, tumor size, tumor necrosis, LVI, hemoglobin, AKP, and LDH variables; multivariate analysis for these inflammation-based prognostic scores was then conducted.

### Comparison of the discriminatory abilities of AGR and established inflammation-based prognostic scores

**Figure 2:**
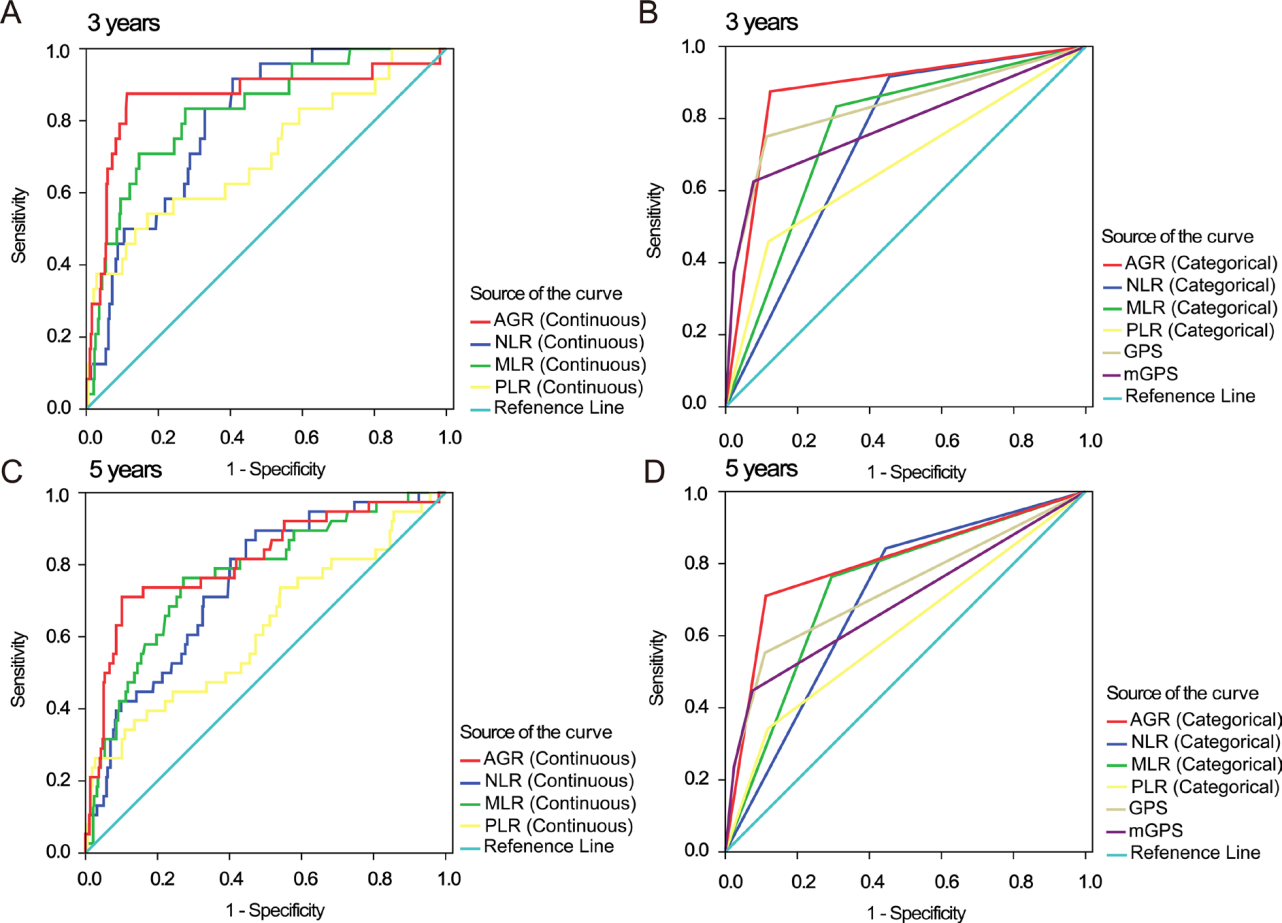
ROC curves for inflammation-based 3- and 5-year prognostic scores ROC curves for AGR, NLR, MLR, PLR (continuous and categorical), GPS, and mGPS for 3- and 5-year survival. Abbreviations: AGR, albumin to globulin ratio; NLR, neutrophil to lymphocyte ratio; MLR, monocyte to lymphocyte ratio; PLR, platelet to lymphocyte ratio; GPS, Glasgow Prognostic Score; mGPS, modified Glasgow Prognostic Score.

To assess the discriminatory ability of AGR compared to that of the established inflammation-based prognostic scores, we generated ROC curves for OS at the 3- and 5-year follow-up examinations, (Figure 2). Table 4 shows a comparison of the discriminatory ability of AGR to that of the other inflammation-based prognostic scores. To ensure that comparisons were rational, continuous indices were compared to continuous AGR data, while categorical indices were compared to the high and low AGR categories. While the area under the curve (AUC) value tended to be higher for AGR than for the other inflammation-based prognostic scores at both 3 years and 5 years, this difference was only significant for some of the prognostic scores (Table 3).

**Table 4:** Comparison of the discriminatory ability of inflammation-based prognostic scores in primary cohort patients before PSM

Period	AUC	95% CI	*p* value	*p* value for comparison to AGR§
**3-year** (continuous)				
AGR (continuous)	0.866	0.768–0.965	< 0.001*	–
NLR (continuous)	0.799	0.727–0.871	< 0.001*	0.246
MLR (continuous)	0.828	0.745–0.911	< 0.001*	0.458
PLR (continuous)	0.705	0.584–0.826	0.001*	0.028*
**3-year** (categorical)				
AGR (categorical)	0.875	0.797–0.954	< 0.001*	–
NLR (categorical)	0.731	0.648–0.814	< 0.001*	0.004*
MLR (categorical)	0.763	0.671–0.855	< 0.001*	0.024*
PLR (categorical)	0.670	0.543–0.797	0.005*	< 0.001*
GPS	0.831	0.726–0.936	< 0.001*	0.239
mGPS	0.781	0.661–0.901	< 0.001*	0.047*
**5-year** (continuous)	.			
AGR (continuous)	0.812	0.730–0.893	< 0.001*	–
NLR (continuous)	0.747	0.672–0.823	< 0.001*	0.224
MLR (continuous)	0.768	0.688–0.849	< 0.001*	0.371
PLR (continuous)	0.621	0.518–0.724	0.014*	0.003*
**5-year** (categorical)				
AGR (categorical)	0.799	0.712–0.886	< 0.001*	–
NLR (categorical)	0.699	0.620–0.777	< 0.001*	0.060
MLR (categorical)	0.734	0.651–0.817	< 0.001*	0.186
PLR (categorical)	0.612	0.509–0.715	0.023*	< 0.001*
GPS	0.728	0.628–0.828	< 0.001*	0.090
mGPS	0.690	0.585–0.794	< 0.001*	0.010*

*p < 0.05.

§AUC values for AGR and for other inflammation-based prognostic scores were compared using MedCalc software. Continuous indexes were compared with continuous AGR scores, while categorical indexes were compared with AGR score categories.

Abbreviations: PSM, propensity score matching; AUC, area under the curves; NLR, neutrophil to lymphocyte ratio; MLR, monocyte to lymphocyte ratio; PLR, platelet to lymphocyte ratio; GPS, Glasgow Prognostic Score; mGPS, modified Glasgow Prognostic Score.

### Predictive nomogram for OS

**Figure 3:**
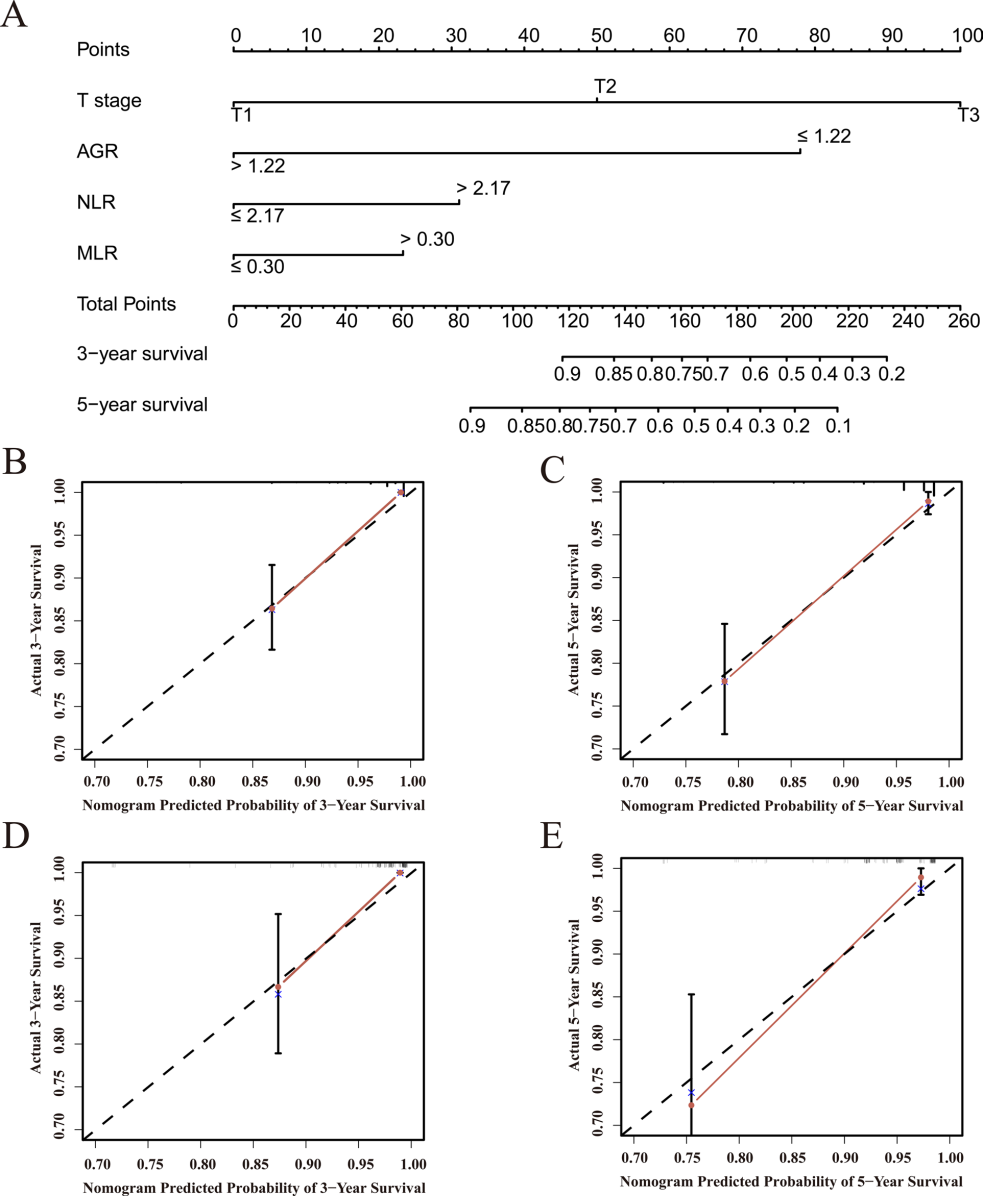
Overall survival predictions based on a nomogram including inflammation-based prognostic scores and clinicopathological characteristics in patients with localized or locally advanced CCRCC (**A**) Nomogram for predicting 3- and 5-year OS in primary cohort patients. Calibration curves for predicting 3- (**B**) and 5-year (**C**) survival in primary cohort patients. Calibration curves for predicting 3- (**D**) and 5-year (**E**) survival in validation cohort patients.

Multivariate analysis indicated that T stage, N stage, AGR, NLR, MLR, GPS, and mGPS were independent risk factors for OS (Table 3); these scores became candidates for inclusion in the final model. Ultimately, the optimal nomogram integrated T stage, AGR, NLR, and MLR to predict 3- and 5-year OS (Figure 3A). The initial concordance index (C-index) values of the Leibovich score [13], SSIGN score [14] and TNM stage of 0.847, 0.838, and 0.806, respectively, increased to 0.899, 0.898, and 0.884 when the AGR (C-index 0.783) was incorporated. The C-index for the nomogram was 0.914, indicating that it had a better predictive ability than Leibovich score (*P* < 0.001), SSIGN score (*P* < 0.001), and TNM stage (*P* < 0.001). Calibration plots for the probability of survival at 3 or 5 years after surgery demonstrated virtually no departures from ideal predictions, confirming the internal validity of the results (Figure 3B, 3C).

### Validation of predictive accuracy of the nomogram for OS

Calibration curves revealed that predictions of 3- and 5-year survival probability obtained using the nomogram were highly consistent with the actual patient OS values (Figure 3D, 3E). The C-index for OS predictions generated using the nomogram was 0.895 in the validation cohort; this was again higher than the C-index values of the Leibovich score (0.808, *P* < 0.001), SSIGN score (0.819, *P* < 0.001), and TNM stage (0.771, *P* < 0.001).

## DISCUSSION

In this study, we analyzed the prognostic power of AGR and the ability of a nomogram that combined AGR with other inflammation-based prognostic scores and clinicopathological characteristics to predict 3- and 5-year survival in patients with localized or locally advanced CCRCC. We found that AGR was an independent prognostic factor for OS and CSS and had a better discriminatory ability than the established inflammation-based prognostic scores. Furthermore, the nomogram that integrated AGR with T stage, NLR, and MLR predicted OS with an accuracy of 0.914.

In recent years, the relationship between AGR, which is a classic indicator of liver function, and cancer has received increasing attention. Research has shown that low AGR is correlated with tumor incidence and mortality rates among healthy people [15], and that AGR is associated with prognosis in breast [9], colorectal [10], nasopharyngeal [11] and lung cancer patients [12]. To our knowledge, the present study is the first to analyze the association between AGR and survival in CCRCC patients.

Several potential mechanisms involved in nutrition and inflammation might contribute to the prognostic value of AGR in cancer. Malnutrition is relatively common in cancer patients, and it often develops into cancer cachexia, which accelerates disease progression. Inflammation associated with tumors involves not only inflammatory factors generated by tumor cells, but also those released during the processes of tissue remodeling and rehabilitation and angiogenesis [16]. Consequently, in addition to changes in the local tumor microenvironment, changes in levels of peripheral inflammatory factors, such as tumor necrosis factor (TNF), interleukin (IL)-1, -6, and -8, and vascular endothelial growth factor (VEGF), also promote tumor growth and metastasis [16–18]. Thus, malignant tumor progression promotes malnutrition and increases inflammation, and vice versa. Effective, practical indicators of nutritional and inflammation status may therefore be useful not only for quantifying tumor malignancy, but also for evaluating patient prognosis. AGR may be a particularly useful indicator in this regard, as it combines nutritional and inflammatory indices. Generally, serum albumin level is thought to reflect the body's nutritional status, and recent studies have shown that it is also an effective indicator of inflammatory state [6, 19, 20]. Serum globulin plays an important role in the body's immune and inflammatory responses as well. High serum globulin levels result from the accumulation of acute-phase proteins and immunoglobulins, and increased expression of these proteins is indicative of a persistent inflammatory state [10]. By combining the albumin and globulin indicators in a single measurement, AGR more accurately reflects the body's nutritional and inflammatory states and may be particularly useful as an outcome indicator for cancer patients.

Previous studies have demonstrated that established inflammation-based prognostic scores, such as NLR, MLR, PLR, GPS and mGPS, are effective in assessing the prognosis of RCC patients [21–25]. Here, we compared the prognostic ability of AGR to that of other established inflammation-based prognostic scores in RCC patients. Generally, as shown in Table 3, the prognostic ability of AGR was superior to that of the established inflammation-based prognostic scores.

Usually, outcome predictions for RCC patients are based on clinicopathological characteristics, such as the TNM staging system and the Fuhrman grading system, and few existing models can successfully predict survival of patients with localized or locally advanced CCRCC. Here, we identified a nomogram that incorporated inflammation-based prognostic scores and traditional staging systems to predict 3- and 5-year OS in these patients more accurately than Leibovich score, SSIGN score, and TNM stage. The nomogram was equally effective in predicting outcomes in the validation cohort patients, and calibration plots for both the primary and validation cohorts revealed that the predicted survival probabilities were closely correlated with the actual survival rates. The well-established Leibovich and SSIGN scores incorporate multiple clinical and pathologic variables, including T stage, N stage, M stage, tumor size, Fuhrman grade, and necrosis. Here, we evaluated the prognostic value of all of these variables individually and found that tumor size, Fuhrman grade, and necrosis were not independent prognostic factors in our patient cohort, suggesting that the two classical prognostic scores may not be useful for patients with localized or locally advanced CCRCC. The incorporation of inflammation-based prognostic scores in addition to clinicopathological characteristics in our nomogram likely enhanced its predictive accuracy. Moreover, AGR, NLR, and MLR can be determined from routine peripheral blood tests conducted during preoperative examinations. The nomogram used here may therefore serve as a reliable tool for predicting survival in patients with localized or locally advanced CCRCC, as well as for selecting patients for adjuvant therapy trials.

Some limitations should be considered when interpreting the results of this study. First, the relatively small number of patients involved were enrolled at a single institution; the results should therefore be verified in a larger, standardized group of patients. In addition, because this was a retrospective study, there were methodological differences among the studies examined, and a multicenter prospective study should be conducted to confirm our findings.

In conclusion, we evaluated the prognostic value of AGR in patients with localized or locally advanced CCRCC for the first time. AGR was an independent prognostic factor and predicted prognosis more accurately than more established inflammation-based prognostic scores. Finally, the incorporation of AGR with inflammation-based prognostic scores and traditional staging systems in a single nomogram increased its predictive accuracy in these CCRCC patients.

## MATERIALS AND METHODS

### Patients

Written informed consent was obtained from all patients enrolled in this study. The study was approved by the Ethics and Scientific Committees of The Third Affiliated Hospital of Soochow University. Clinicopathological data were collected from 592 patients with RCC who underwent radical or partial nephrectomy in The Third Affiliated Hospital of Soochow University (China) between 2003 and 2013. To ensure that data were collected objectively and accurately, the following exclusion criteria were used: patients with a history of anti-tumor therapy and other concurrent tumors; other acute or chronic concurrent non-cancer diseases (including liver disease, inflammation, and infection); concurrent distant metastasis; patients lost to follow-up. 176 patients who were enrolled between May 2012 and December 2013 were assigned to the external validation cohort; all other patients were assigned to the primary cohort.

### Data collection

The following clinicopathologic data were collected for each enrolled patient: age at surgery, sex, tumor size, Fuhrman grade, and the presence or absence of tumor necrosis, lymph node invasion, and lymphovascular invasion. TNM stage was assigned according to the 2010 AJCC TNM classification [3]. Tumor necrosis was defined as microscopic coagulative necrosis [26]. Lymphovascular invasion was defined as tumor cell invasion of blood vessels or lymphatic vessels, but not the underlying muscular walls [27]. Relevant laboratory indicators and levels of C- reactive protein (CRP), albumin, globulin, Hb, LDH, etc., were collected one week before surgery. Post-operative follow-ups occurred every six months for the first three years and annually thereafter for locally advanced CCRCC patients. For localized CCRCC patients, follow-up imaging was performed twice in the first year and annually thereafter. No neoadjuvant or adjuvant treatments were administered. AGR was calculated as AGR = albumin / (total protein - albumin). NLR is the ratio of neutrophils to lymphocytes [21]; MLR is the ratio of monocytes to lymphocytes [22]; and PLR is the ratio of platelets to lymphocytes [23]. GPS was classified as follows: score 2 if serum CRP > 10 mg/L and albumin < 35 g/L; score 1 if CRP > 10 mg/L or albumin < 35 g/L; score 0 if serum CRP ≤ 10mg/mL and albumin > 35 g/L [24]. mGPS was classified as follows: score 2 if serum CRP > 10 mg/L and albumin < 35 g/L; score 1 if serum CRP > 10 mg/L and albumin ≥ 35 g/L; score 0 if CRP ≤ 10 mg/L [28].

### Statistical analysis

The optimal cut-off values were determined using receiver operating characteristics (ROC) curve analysis. OS rates were calculated using the Kaplan–Meier method and compared to detect statistically significant differences using the log-rank test. PSM was conducted using a nearest-neighbor matching algorithm with a maximum tolerated difference between propensity scores of less than 30% of the propensity score SD. Mantel-Cox regression methodology was used for univariate analysis of implicit factors affecting survival. Only variables with *P* < 0.05 in univariate analyses were included in the multivariate Cox's proportional hazards model. AUC values of the ROC curves were compared to evaluate the discriminatory ability of AGR and the other established inflammation-based prognostic scores in the assessing prognosis. Differences were compared with MedCalc software (Version 11.4.2.0, MedCalc, Inc., Belgium) to determine whether they were statistically significant.

The nomogram was constructed based on the multivariate analysis results. To find a best-fit model, backward stepwise selection with the Akaike information criterion (AIC) was used in a Cox proportional hazards regression model [29]. Both discrimination and calibration were used to evaluate nomogram performance. The c-index and the ROC curves were used to compare OS discrimination ability among different models. Confidence intervals (CIs) were obtained by creating 500 bootstrap samples from the entire data set and replicating the estimation process.

Statistical tests were performed using SPSS software (SPSS 22.0, Chicago, IL, USA), MedCalc software, and R software version 3.2.3 (http://www.r-project.org/) with Hmisc, rms, and survival ROC packages. Two-sided *P* values < 0.05 were considered statistically significant.
